# Persistent Exhausted T-Cell Immunity after Severe COVID-19: 6-Month Evaluation in a Prospective Observational Study

**DOI:** 10.3390/jcm12103539

**Published:** 2023-05-18

**Authors:** Elena Vazquez-Alejo, Laura Tarancon-Diez, Maria de la Sierra Espinar-Buitrago, Miguel Genebat, Alba Calderón, Guillermo Pérez-Cabeza, Esmeralda Magro-Lopez, Manuel Leal, Mª Ángeles Muñoz-Fernández

**Affiliations:** 1 Immunology Section, Molecular Immuno-Biology Laboratory, Hospital General Universitario Gregorio Marañón, Instituto de Investigación Sanitaria Gregorio Marañón (IiSGM), 28007 Madrid, Spain; elena2393.ev@gmail.com (E.V.-A.); ltarancondiez@gmail.com (L.T.-D.); marisierri90@gmail.com (M.d.l.S.E.-B.); esmeraldamagrolopez@hotmail.com (E.M.-L.); 2 Centro de Investigación Biomédica en Red de Bioingeniería, Biomateriales y Nanomedicina (CIBER-BBN), Instituto de Salud Carlos III, 28029 Madrid, Spain; 3 Internal Medicine Department, Hospital Fátima, 41012 Sevilla, Spain; mgenebat@yahoo.es (M.G.); alba.cal.pec@gmail.com (A.C.); 4 Urgency Department, Hospital Viamed Santa Ángela de la Cruz, 41013 Sevilla, Spain; gperez@viamedsalud.com; 5 Internal Medicine Department, Hospital Viamed Santa Ángela de la Cruz, 41013 Sevilla, Spain; mleal@telefonica.net

**Keywords:** SARS-CoV-2, severe COVID-19, hospitalization, immune exhaustion profile, SARS-CoV-2-specific T-cell response, long-term evolution

## Abstract

Introduction: Severe COVID-19 can result in a significant and irreversible impact on long-term recovery and subsequent immune protection. Understanding the complex immune reactions may be useful for establishing clinically relevant monitoring. Methods: Hospitalized adults with SARS-CoV-2 between March/October 2020 (n = 64) were selected. Cryopreserved peripheral blood mononuclear cells (PBMCs) and plasma samples were obtained at hospitalization (baseline) and 6 months after recovery. Immunological components’ phenotyping and SARS-CoV-2-specific T-cell response were studied in PBMCs by flow cytometry. Up to 25 plasma pro/anti-inflammatory cytokines/chemokines were assessed by LEGENDplex immunoassays. The SARS-CoV-2 group was compared to matched healthy donors. Results: Biochemical altered parameters during infection were normalized at a follow-up time point in the SARS-CoV-2 group. Most of the cytokine/chemokine levels were increased at baseline in the SARS-CoV-2 group. This group showed increased Natural Killer cells (NK) activation and decreased CD16^high^ NK subset, which normalized six months later. They also presented a higher intermediate and patrolling monocyte proportion at baseline. T cells showed an increased terminally differentiated (TemRA) and effector memory (EM) subsets distribution in the SARS-CoV-2 group at baseline and continued to increase six months later. Interestingly, T-cell activation (CD38) in this group decreased at the follow-up time point, contrary to exhaustion markers (TIM3/PD1). In addition, we observed the highest SARS-CoV-2-specific T-cell magnitude response in TemRA CD4 T-cell and EM CD8 T-cell subsets at the six-months time point. Conclusions: The immunological activation in the SARS-CoV-2 group during hospitalization is reversed at the follow-up time point. However, the marked exhaustion pattern remains over time. This dysregulation could constitute a risk factor for reinfection and the development of other pathologies. Additionally, high SARS-CoV-2-specific T-cells response levels appear to be associated with infection severity.

## 1. Introduction

COVID-19 disease often manifests in an asymptomatic/oligosymptomatic form. However, up to 15% of infected patients have a severe clinical presentation that can progress to acute respiratory distress syndrome and severe pathologies necessitating hospitalization, which may result in mortality. In adults, disease severity is linked to specific risk factors, such as age and/or comorbidities, including diabetes mellitus and hypertension, among others [1,2].

The inflammation caused by SARS-CoV-2 has been extensively studied, and the primary cause is reported to be an exacerbated immune response in patients in the form of a proinflammatory cytokines storm, leading to depletion and alteration in innate and adaptive immunity components [3,4,5]. In the innate immunity arm, an altered pattern of monocytes, natural killer (NK), and dendritic cells has been observed in the context of SARS-CoV-2, which results in a series of inefficient immune responses [6,7,8]. However, their role in the immunopathogenesis and short- and long-term sequelae of COVID-19 requires further elucidation. In addition, SARS-CoV-2 alters the distribution and functionality of CD4 and CD8 T-cell subsets, which are essential for an effective adaptive immune response, and this immune disturbance has been correlated with a worse prognosis in the disease progression [5,9].

As demonstrated, the host immune response to SARS-CoV-2 infection plays a critical role in the disease’s progression, and an imbalance in this response can be fatal [10]. In the case of severe COVID-19 requiring hospitalization, the restoration of immune function after severe infection may be crucial, as it is necessary and would indicate the immune system’s readiness and ability to respond to future new infections or vaccine administrations. It is still unclear whether severe infection could have a significant and irreversible impact on short- and long-term recovery and subsequent immune protection. Thus, in addition to a combination of biomarkers for accurately predicting COVID-19 clinical outcomes, identifying immune biomarkers that could also aid in predicting immune function recovery during the immediate follow-up is needed. To achieve this, we assessed the biochemical, immune phenotypic, and functional status of patients presenting with clinically severe COVID-19 between March and October 2020, and six months after recovery, with a specific focus on inflammation and immune exhaustion in both innate and adaptive immune compounds, and compared them with healthy controls. 

## 2. Materials and Methods

### 2.1. Study Participants and Design

Patients who were diagnosed with SARS-CoV-2 infection by positive RT-PCR at Hospital Fátima and Hospital VIAMED Santa Ángela de la Cruz in Sevilla (Spain) between March and October 2020, were enrolled in the study based on the following inclusion criteria: (1) hospitalization due to SARS-CoV-2-related pneumonia; and (2) availability of clinical, immune, and biochemical data for hospital admission, hospitalization (baseline), hospital discharge, and follow-up (6 months later). Inclusion in the study required data for at least three of those four time points. The study group, referred to as the SARS-CoV-2 group (n = 64), was compared to a reference group of non-SARS-CoV-2 infected individuals (previous negative for SARS-CoV-2 RT-PCR and SARS-CoV-2 anti-IgM/IgG) matched by sex and age, called the HEALTHY DONORS group (n = 25), recruited during the same time period. 

The study was approved by the Ethics Committee of Hospital General Universitario Gregorio Marañón (HGUGM) and the participant hospitals, and written informed consent was obtained from all SARS-CoV-2 patients and healthy donors prior to inclusion in the study.

### 2.2. Clinical Data and Laboratory Determinations

Clinical and epidemiological data of SARS-CoV-2 infected patients were collected from their medical records by the Internal Medicine Departments of the participating hospitals. 

Longitudinal clinical variables, such as length of hospitalization, clinical outcomes, immunological and biochemical parameters, and the presence of comorbidities were documented throughout the study. Additionally, 30 mL samples of fresh whole blood were collected in ethylene diamine tetra-acetic acid (EDTA) tubes from both SARS-CoV-2 patients and healthy donors at baseline, and from the SARS-CoV-2 group 6 months later. Plasma and cryopreserved peripheral blood mononuclear cells (PBMCs) were immediately isolated using Ficoll–Paque density gradient centrifugation and stored at −20 °C and −170 °C, respectively, in the Spanish HIV-HGM BioBank until use.

### 2.3. Cytokine and Chemokine Quantification

Up to 13 pro/anti-inflammatory soluble cytokines and 12 additional chemokines were quantified on plasma from healthy donors and on hospitalization time point and 6 months’ time point from SARS-CoV-2 group using Cytokine Panel 1 and Proinflammatory Chemokine Panel 1, respectively, from LEGENDplex immunoassays and Qognit software (both from BioLegend, San Diego, CA, USA) and CytoFlex S flow cytometer (Beckman Coulter, Brea, CA, USA).

### 2.4. Cell Immunophenotyping 

Immunophenotyping of T cells, NK cells, and monocytes was performed using multiparametric flow cytometry in batches consisting of 5 SARS-CoV-2 patients paired with 3 healthy donors per assay, following this cellular hierarchy order according to sample availability. The number of patients included in each subanalysis during this study may vary due to this limitation.

As general protocol and as we have described previously [11], PBMCs were thawed and resuspended in RPMI media supplemented with 10% heat-inactivated fetal bovine serum (FBS), 100 U/mL penicillin G and 100 µL/mL streptomycin sulfate and washed with phosphate-buffered saline (PBS) containing 3% of bovine serum albumin (BSA). FC receptors blocking antibodies were not used to block non-specific binding [12]. Then, cells were incubated with LIVE/DEAD fixable Aqua Blue Dead Cell Stain (Life Technologies, Carlsbad, CA, USA) for viability for 5 min at room temperature (RT) before staining. 

For T cells, PBMCs were then stained with surface antibodies markers for 35 min at RT with Brilliant Stain Buffer (BD Biosciences, Franklin Lakes, NJ, USA), including cell viability, lineage (CD14, CD19, CD56, CD3, CD4 and/or CD8), maturation (CD45RA, CD27), activation (CD38, CD137, CD154, HLA-DR), senescence (CD57), exhaustion (TIM3, LAG3, TIGIT, PD1), recent thymic emigrants (CD31), IL-7 (CD127) and IL-2 (CD25) receptors’ markers. Then, cells were permeabilized using Foxp3/Transcription Factor Staining Buffer (Ebioscience, San Diego, CA, USA) according to the manufacturer’s protocol, stained for 30 min at 4 °C with the intranuclear transcription marker (FoxP3) for T regulatory cells (Treg) characterization and fixed for 20 min at 4 °C with 4% paraformaldehyde (PFA) solution. These markers were distributed into three different T-cell panels described in detailed at Supplementary Table S1 (data available for 31 SARS-CoV-2 patients and 18 healthy donors). T cells were gated based on CD3, and/or CD8/CD4, but not CD19, CD14, and CD56; and T-cell maturation subset based on CD45RA and CD27 expression as naïve (CD45RA^+^CD27^+^), central memory (CM; CD45RA^−^CD27^+^), effector memory (EM; CD45RA^−^CD27^−^) and terminally differentiated (TemRA; CD45RA^+^CD27^−^) T cells. Treg were defined by CD4 and FoxP3/CD25 co-expression. Isotype controls were used to determine the expression of immunophenotyping markers. Schematic gating strategy for T cells can be found in Supplementary Figure S1A. 

For NK cells (data available for 31 SARS-CoV-2 patients and 18 healthy donors), thawed PBMCs were surface stained with cell viability marker and antibodies for linage (CD3, CD14, CD19, CD56, and CD16); activation (HLA-DR and CD158b); maturation (CD57 and TIM-3); and C-type lectin-like activating and inhibiting receptors (NKG2D and NKG2A, respectively) for 35 min at RT with Brilliant Stain Buffer and fixed for 20 min at 4 °C with 4% PFA solution (detailed description at Supplementary Table S1). Viable NK cells were classified into three subsets according to the expression of CD56 and CD16: CD56^high^, CD56^dim^ (that includes the CD16^high^ subset), and CD56^neg^ subsets. The expression of markers for immunophenotyping was determined using isotype controls. Representative gating strategy can be found in Supplementary Figure S1B. 

For monocytes (data available for 31 SARS-CoV-2 patients and 18 healthy donors), thawed PBMCs were stained with cell viability and surface markers for linage and monocytes’ subsets identification (CD3, CD19, CD56, HLA-DR, CD14 and CD16); endothelial cell adhesion (CD11b, CD62L, and CD49d); and activation (CD40) for 35 min at RT with Brilliant Stain Buffer and fixed for 20 min at 4 °C with 4% PFA solution (Supplementary Table S1). Monocytes were defined as viable cells negative for CD56, CD19, and CD3 and positive for HLA-DR and CD14. Isotype controls were used to determine the expression of immunophenotyping markers. Schematic gating strategy for monocytes can be found in Supplementary Figure S1C.

### 2.5. SARS-CoV-2-Specific Cellular T-Response

SARS-CoV-2-specific T-cell response was assessed in vitro after SARS-CoV-2 pool of peptides stimulation originated from structural (S, M, N, E) and non-structural viral proteins. Thawed PMBCs were resuspended in RPMI media, supplemented with 10% heat-inactivated calf serum, 100 U/mL penicillin G, and 100 µL/mL streptomycin sulfate, and rested for 2 h with 10 U/mL DNase I (Roche Diagnoses, Basel, Switzerland). Then cells were incubated for 6 h at 37 °C/5% CO_2_ in the presence or absence of 1 µg/mL of SARS-CoV-2 peptides (Miltenyi Biotec, Bergisch Gladbach, Germany) and with 10 µg/mL of anti-CD28/anti-CD49d, 1 µg/mL of Golgy stop (BD Biosciences), 2 µg/mL of Brefeldin A (Biolegend, San Diego, CA, USA) and CD107a surface degranulation marker. 

Then, PBMCs were washed with PBS 3% of BSA and surface staining for T cells was performed using LIVE/DEAD fixable Aqua Blue Dead Cell Stain for viability; CD3 and CD4 or CD8 for linage; CD45RA and CD27 for memory subset identification for 35 min at RT with Brilliant Stain Buffer. Then cells were permeabilized and fixed using a Cytofix/Cytoperm kit (BD Biosciences) for 35 min at 4 °C following the manufacturer’s protocol and stained intracellularly with CD3, TNF-α, IFN-γ, and IL2 for 35 min at 4 °C, and fixed for 20 min at 4 °C with 4% PFA solution (detailed description at Supplementary Table S1) (data available for 22 SARS-CoV-2 patients). Representative gating strategy for specific T-cell response can be found in Supplementary Figure S1D.

SARS-CoV-2 T-cell response analyses included background subtraction using an unstimulated condition as negative control and a positive control stimulated with staphylococcal enterotoxin B (SEB, Sigma Aldrich, St Louis, MO, USA). Cell acquisition was carried out in a Gallios flow cytometer (Beckman Coulter). Before acquisition, cells were fixed for 20 min with 4% PFA. At least 1 million events were acquired for each condition. FlowJo V10 software (TreeStar, Woodburn, OR, USA) was used for data analysis.

### 2.6. Statistical Analysis

The Statistical Package for the Social Sciences software (SPSS 20.0, Chicago, IL, USA) was used for the statistical analysis. Graphs were generated using GraphPad Prism 9.0 (GraphPad Software, Inc., San Diego, CA, USA). Continuous variables were expressed as median and interquartile range (IQR). Categorical variables were expressed as number and percentage. Differences between categorical and continuous values were determined using the chi-square test and two-tailed Mann–Whitney *U*-test, respectively. Wilcoxon matched-pairs signed-rank test was conducted to compare paired samples. The Spearman’s rank test was used to analyze correlations between variables. *p*-values < 0.05 were considered statistically significant.

## 3. Results

### 3.1. Baseline Clinical Characteristics of the Studied Subjects

Epidemiological and clinical data of the 64 SARS-CoV-2 patients are in Table 1. The median age was 57 years, and 23 (36%) were women. All presented severe COVID-19-related symptoms and required hospitalization and high-flow supplementary oxygen during admission. The median hospitalization time was 7 days, and the median duration of symptoms was 16 days. Six subjects died during the study due to SARS-CoV-2 pneumonia. Of note, 15 subjects had hypertension, and 7 were diabetic. All patients received standard pharmacological antiviral treatment and glucocorticoid therapy approved for SARS-CoV-2 infection during the study.

### 3.2. Longitudinal Analysis of Biochemical and Hematological Data of SARS-CoV-2 Patients

A four-point evolution analysis (at hospital admission, hospitalization (baseline), hospital discharge, and follow-up (6 months later)) of biochemical and hematological parameters was performed.

**Figure 1:**
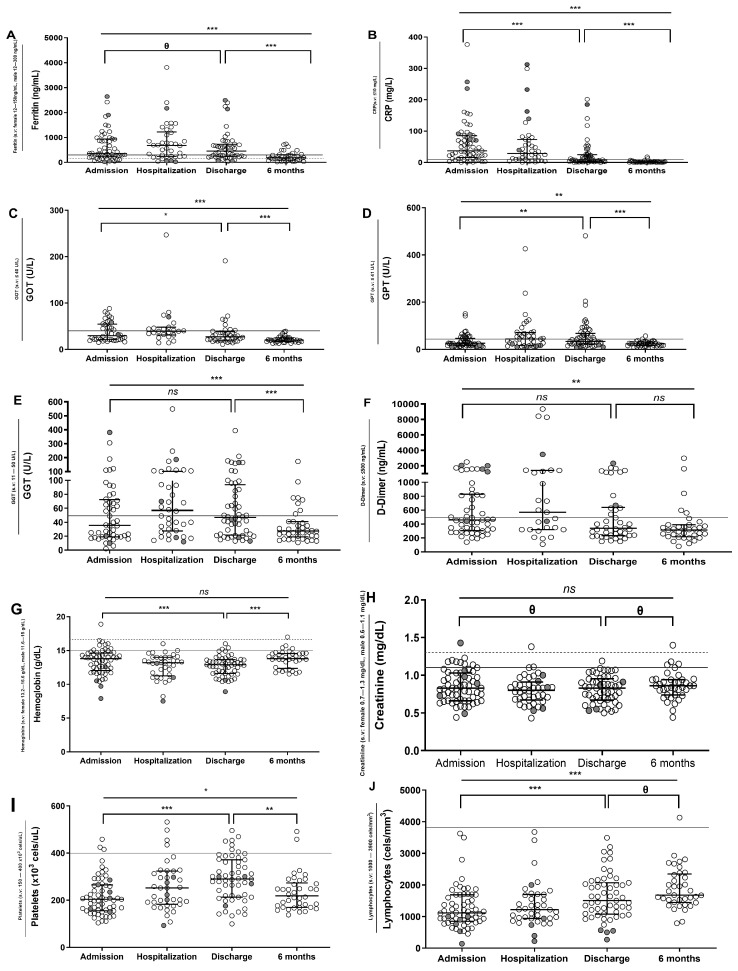
Evolution of biochemical and hematological parameters in SARS-CoV-2 group. Longitudinal analysis at admission (data available for 62 patients), hospitalization (data available for 40 patients), discharge (data available for 55 patients), and after 6 months (data available for 40 patients) of ferritin, C-reactive protein (CRP), glutamic oxalocetic transaminase (GOT), glutamic pyruvic transaminase (GPT), gamma glutamyl transferase (GGT), D-dimer, hemoglobin, creatinine, platelets, and lymphocytes levels from SARS-CoV-2 group (**A**–**J**). Gray dashed/continue line is the upper reference limit from which an abnormal value is considered for female and male, respectively. Six patients died during the study and are highlighted in gray. Abbreviations: s.v: standard values. Lines represent medians and interquartile ranges. Wilcoxon test was conducted to compare paired events. *** *p* ≤ 0.001, ** *p* ≤ 0.01, * *p* < 0.05, *Ɵ* 0.05 ≤ *p* ≤ 0.1, *ns p* > 0.1.

Ferritin, C-reactive protein (CRP), transaminases (glutamic oxaloacetic transaminase (GOT) and glutamic pyruvic transaminase (GPT)), hepatic enzyme gamma glutamyl transferase (GGT), and D-dimer levels increased during the acute viral phase at hospitalization, decreased in the discharge, and continued to reduce six months later (Figure 1A–F). Hemoglobin levels decreased during hospitalization but showed an increment after six months (Figure 1G). Creatinine did not show significant differences during the study (Figure 1H). Platelet levels increased during hospitalization and decreased six months later (Figure 1I), in contrast to the evolution of lymphocytes (Figure 1J).

### 3.3. Different Soluble Cytokine and Chemokine Levels in SARS-CoV-2 Subjects

**Figure 2:**
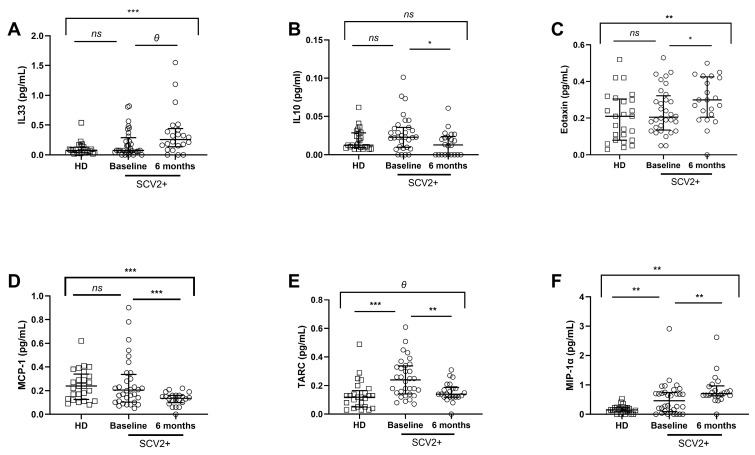
Soluble pro/anti-inflammatory cytokine and chemokine levels in plasma. Differences at baseline and 6 months later from the SARS-CoV-2 group (data available for 32 and 22 SARS-CoV-2 patients, respectively) and healthy donors (data available for 18 subjects). Soluble IL-33, IL-10, Eotaxin, MCP-1, TARC, and MIP-1α levels’ plasma at baseline and 6 months later (**A**–**F**). SCV2+ are highlighted with white dots, and white squares represent HD. Lines represent medians and interquartile ranges. Mann–Whitney U-test was used for group comparisons. Wilcoxon test was conducted to compare paired events in the SARS-CoV-2 group. SCV2+, SARS-CoV-2 group; HD, Healthy Donors. *** *p* ≤ 0.001, ** *p* ≤ 0.01, * *p* < 0.05, *Ɵ* 0.05 ≤ *p* ≤ 0.1, *ns p* > 0.1.

The analysis of soluble cytokines/chemokines (Supplementary Table S2) showed that no differences were found in the majority of cytokines. In the case of IL-33, levels increased over time in SARS-CoV-2 subjects compared to healthy donors, in contrast to IL-10 levels, which decreased in the SARS-CoV-2 group six months later (Figure 2A,B).

In general, chemokines were increased at baseline in the SARS-CoV-2 group compared to healthy donors, but after 6 months, altered levels were only recovered in some chemokines. Despite no significant differences being found comparing Eotaxin and MCP-1 between both groups at baseline, Eotaxin levels significantly increased after six months in SARS-CoV-2 patients compared to healthy donors, and MCP-1 levels decreased (Figure 2C,D). In the case of TARC and MIP-1α, levels were higher in SARS-CoV-2 patients at the baseline; nevertheless, TARC levels significantly reduced, and MIP-1α levels continued to increase 6 months later in these patients (Figure 2E,F).

Curiously, we found correlations between cytokine levels and age and biochemical/hematological data from the SARS-CoV-2 group at baseline (Supplementary Figure S2A). Notably, there was a direct and strong association between IL-33 and hemoglobin levels, IL-10 and age, and IL-18 and ferritin (Supplementary Figure S2B–D). In contrast, we observed an inverse correlation between MCP-1 and GGT levels (Supplementary Figure S2E). Moreover, we found associations between soluble chemokines and age and biochemical/hematological (Supplementary Figure S2F). We determined a direct correlation between Eotaxin and alkaline phosphatase levels, and TARC and age (Supplementary Figure S2G,H), in contrast to IL-8 and ITAC, which correlated inversely with GOT and hemoglobin levels, respectively (Supplementary Figure S2I,J).

### 3.4. SARS-CoV-2 Patients Show Different NK and Monocyte Cell Subsets Distribution and Increased Expression of Activation and Endothelial Adhesion Markers

**Figure 3:**
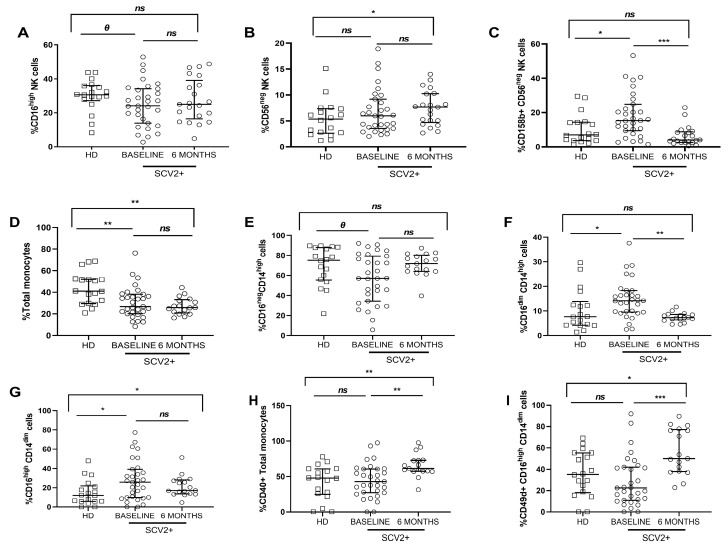
Frequency of NK and monocyte cell subsets and activation and endothelial adhesion markers expression. Differences at baseline and 6 months later from SARS-CoV-2 group and healthy donors. Frequency of the CD16^high^ NK cell subset (**A**); frequency of CD56^neg^ and CD158b expression in the CD56^neg^ NK cell subset (**B**,**C**); frequency of total monocytes, and proportion of classical (CD16^neg^ CD14^high^), intermediate (CD16^dim^ CD14^high^) and patrolling (CD16^high^ CD14^dim^) monocyte subsets (**D**–**G**); CD40, CD49d expression in total monocytes and in the patrolling subsets, respectively (**H**,**I**). SCV2+ are highlighted with white dots, and white squares represent HD. Lines represent medians and interquartile ranges. Mann–Whitney U-test was used for group comparisons. Wilcoxon test was conducted to compare paired events in the SARS-CoV-2 group. SCV2+, SARS-CoV-2 group; HD, Healthy Donors’ group. *** *p* ≤ 0.001, ** *p* ≤ 0.01, * *p* < 0.05, *Ɵ* 0.05 ≤ *p* ≤ 0.1, *ns p* > 0.1.

Regarding NK cell immunophenotyping (Supplementary Table S3), the SARS-CoV-2 group showed lower levels, though not significant, of the CD16^high^ NK cell subset, compared to healthy donors at baseline (Figure 3A). By contrast, the CD56^neg^ NK cell subset and the activation CD158b marker expression in the CD56^neg^ NK subset were increased in SARS-CoV-2 patients at baseline, and interestingly, the proportion of the CD56^neg^ NK cell subset continued to increase significantly six months later, whereas CD158b expression in this NK subset was normalized relative to healthy donors (Figure 3B,C). At baseline, soluble IFN-γ levels from all subjects strongly and directly correlated with the inhibiting receptor NKG2A and maturation CD57 marker in the CD16^high^ NK subset (Supplementary Figure S3A,B). In addition, at baseline, soluble MCP-1 levels were directly associated with NKG2A expression in the CD56^high^ NK cell subset, but these correlations did not persist 6 months later (Supplementary Figure S3C).

Monocyte subsets were defined as classical monocytes (CD16^neg^ CD14^high^), intermediate monocytes (CD16^dim^ CD14^high^), and patrolling monocytes (CD16^high^ CD14^dim^) (Supplementary Table S3). At baseline, SARS-CoV-2 patients showed a lower frequency of total, lower proportion of classical monocytes and a higher proportion of intermediate and patrolling monocytes compared to healthy donors. No changes were observed after six months, except in the intermediate subset, which decreased (Figure 3D–G). At baseline, there were no significant differences in CD40 expression in total monocytes and CD49d in patrolling subsets between both groups, but in both cases, the expression significantly increased at the follow-up time point (Figure 3H,I). Interestingly, a direct significant correlation was found between soluble IL-12p70 from all subjects and total monocytes’ frequency at baseline (Supplementary Figure S3D). Furthermore, six months later, soluble IL-18 levels correlated inversely with the intermediate subset from the SARS-CoV-2 group (Supplementary Figure S3E). Concerning chemokines, MCP-1 correlated strongly and directly with the activation marker CD40 in total monocytes from the SARS-CoV-2 group (Supplementary Figure S3F).

### 3.5. SARS-CoV-2 Patients Show High Levels of Activation and Exhaustion Markers in CD4 and CD8 T Cells

**Figure 4:**
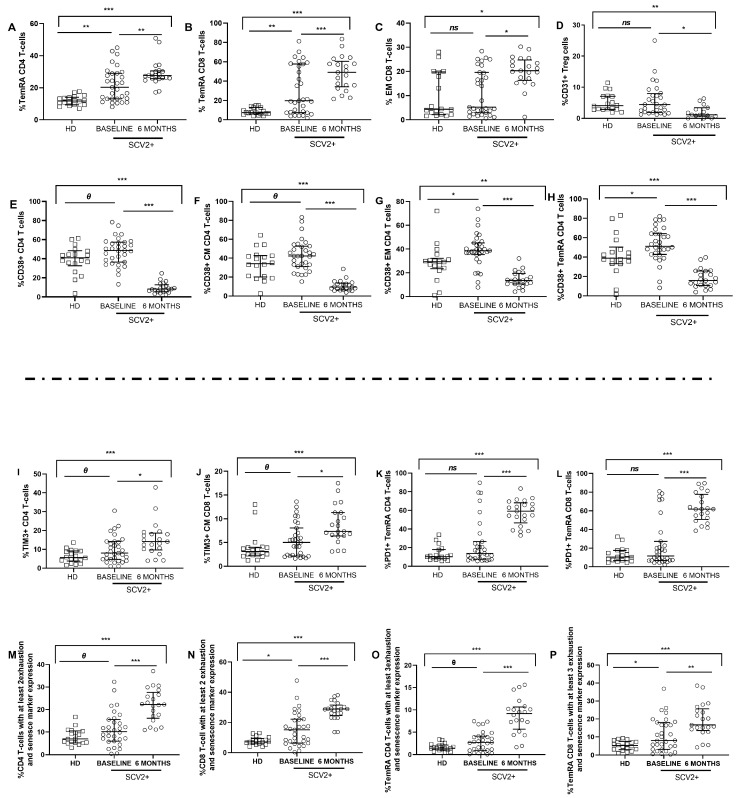
Maturation profile, activation, exhaustion, and senescence markers on CD4 and CD8 T cells and Treg cells. Differences at baseline and 6 months later. Differences in T-cell memory subsets distribution (**A**–**C**); CD31 expression in Treg cells (**D**); activation markers expression in total CD4 T cells and memory subsets (**E**–**H**); TIM3 (**I**,**J**) and PD1 expression (**K**,**L**) in total and memory T cells. Percentage of expression of at least 2 exhaustion and senescence markers (CD57, PD1, TIGIT, and/or TIM3) in CD4 and CD8 T cells (**M**,**N**) and expression levels of at least 3 exhaustion and senescence markers (CD57, PD1, TIGIT, and/or TIM3) in the TemRA CD4 and CD8 T-cells memory subset (**O**,**P**). SCV2+ are highlighted with white dots, and white squares represent HD. Lines represent medians and interquartile ranges. Abbreviations: TemRA, terminally differentiated; EM, effector memory; CM, central memory; SCV2+, SARS-CoV-2 group; HD, healthy donors’ group. Mann–Whitney *U*-test was used for group comparisons. Wilcoxon test was conducted to compare paired events in the SARS-CoV-2 group. *** *p* ≤ 0.001, ** *p* ≤ 0.01, * *p* < 0.05, *Ɵ* 0.05 ≤ *p* ≤0.1, *ns p* > 0.1.

T cells immunophenotyping analysis (Supplementary Table S3) revealed differences in the distribution of maturation subsets, with increased levels of terminally differentiated (TemRA, CD45RA^+^CD27^−^) CD4 and CD8 T cells in SARS-CoV-2 patients compared to healthy donors at baseline, which continued to increase after 6 months (Figure 4 A,B). No significant differences were observed in the naïve (CD45RA^+^CD27^+^) and Central Memory (CM, CD45RA^−^CD27^+^) CD4 and CD8 T-cell memory subsets. Although no significant differences in Effector Memory (EM, CD45RA^−^CD27^−^) CD8 T-cell subset were observed between both groups at baseline, the EM proportion increased after six months (Figure 4C). Interestingly, at baseline, the frequency of Treg expressing the recent thymic emigrant marker CD31 showed no differences between groups, but these levels significantly decreased six months later (Figure 4D).

Regarding activation markers, at baseline, we observed higher expression of CD38 in total and memory CD4 T-cells subsets in the SARS-CoV-2 group compared to healthy donors, although this activation decreased over time (Figure 4E–H). Similar results were obtained for total and CD8 T-cells memory subsets. No significant differences were found in the co-expression of CD38 and HLA-DR markers in T cells. However, at baseline, the levels of HLA-DR expression in CD4 T-cells and CM CD8 T-cell memory subset inversely correlated with soluble IL-10 levels in all the subjects studied (Supplementary Figure S4A,B), and HLA-DR expressed in CM CD4 T cells directly correlated with soluble IL-23 in all subjects (Supplementary Figure S4C), only at baseline. In addition, the levels of CD154 and CD38 in CD4 T cells in all subjects correlated directly with soluble MIP-α also at baseline (Supplementary Figure S4D,E).

The study of exhaustion and senescence in T cells showed increased levels of TIM-3 expression in total CD4 T cells and CM CD8 T cells in SARS-CoV-2 patients compared to healthy donors at baseline and continued to increase after six months (Figure 4I,J). Despite no differences in PD1 expression in TemRA CD4 and CD8 memory subsets being observed at baseline between both groups, PD1 expression significantly increased over time (Figure 4K,L). In the case of correlations with chemokines at 6-months time point, soluble MIG levels directly correlated with PD1 in the TemRA CD8 T-cell memory subset in the SARS-CoV-2 group (Supplementary Figure S4F).

The simultaneous expression of more than one of the examined exhaustion and senescence markers (CD57, PD-1, TIGIT, and TIM-3) was also analyzed. At baseline, SARS-CoV-2 patients showed a higher percentage of expression of at least 2 exhaustion and senescence markers in CD4 and CD8 T cells, and this frequency increased after six months compared to healthy donors (Figure 4M,N). Similar results were obtained in the study for the expression of at least 3 exhaustion and senescence markers in TemRA CD4 and CD8 T-cell memory subsets (Figure 4O,P).

### 3.6. SARS-CoV-2-Specific T-Cell Response after Six Months in Severe SARS-CoV-2 Recovered Patients

**Figure 5:**
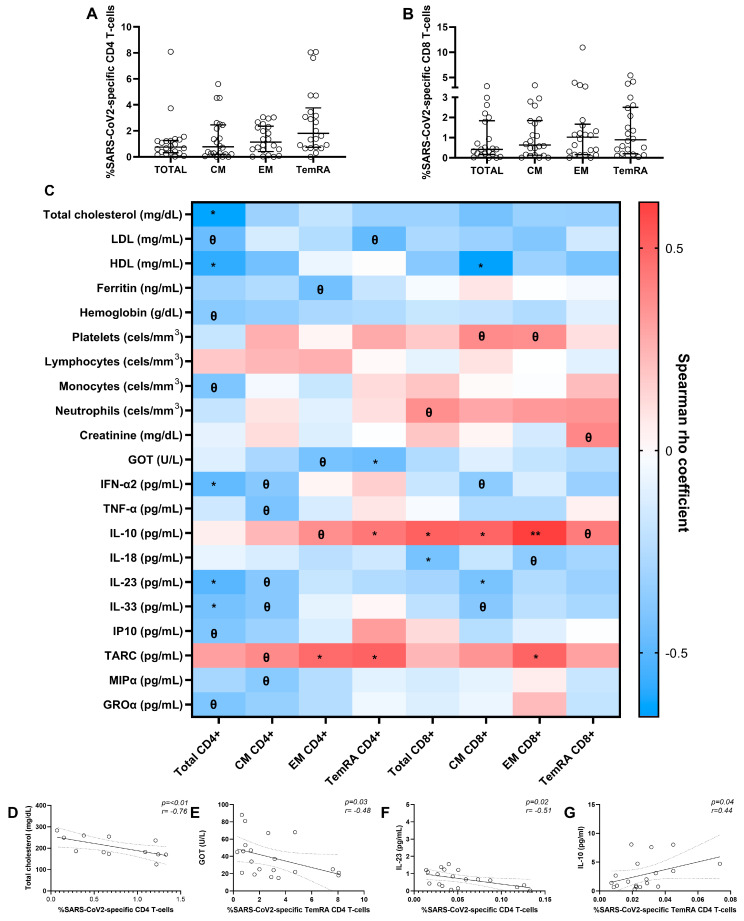
SARS-CoV-2-specific CD4 and CD8 T-cell response in SARS-CoV-2 patients after 6 months and correlations with baseline biochemical, hematological parameters, and soluble cytokines/chemokines. Specific total and memory CD4 and CD8 T-cell subsets (central memory (CM), effector memory (EM), and terminally differentiated (TemRA)) response to SARS-CoV-2 (**A**,**B**). Associations between SARS-CoV-2-specific T-cell response from recovered SARS-CoV-2 patients after six months and biochemical, hematological parameters, and soluble cytokines/chemokines at baseline (**C**). Significant correlations with total cholesterol (data available for 14 patients) (**D**), glutamic oxalocetic transaminase (GOT) (data available for 20 patients) (**E**), soluble IL-23 levels (data available for 21 patients) (**F**), and soluble IL-10 levels (data available for 20 patients) (**G**). Lines represent medians and interquartile ranges. Each dot represents a SARS-CoV-2 patient. The Spearman ρ correlation coefficient test was used (**C**–**G**). ** *p* ≤ 0.01, * *p* < 0.05, *Ɵ* 0.05 ≤ *p* ≤ 0.1.

We assessed specific CD4 and CD8 T-cell responses to SARS-CoV-2 at 6-months time point. The specific T-cell response to the virus was determined by the sum of combinations of studied functions (CD107a, TNF-α, IFN-γ, and IL2). We observed the highest response magnitude in TemRA CD4 T-cell and EM CD8 T-cell memory subsets (Figure 5A,B).

We analysed which altered clinical and inflammatory parameters during the infection may be associated with a greater SARS-CoV-2-specific T-cell response after six months (Figure 5C). Interestingly, we observed that the frequency of SARS-CoV-2-specific CD4 T cells correlated strongly and inversely with total cholesterol levels, and SARS-CoV-2-specific TemRA CD4 T cells negatively correlated with GOT levels (Figure 5D,E). Moreover, a significant inverse correlation between SARS-CoV-2-specific total CD4 T cells and soluble IL-23 levels and a direct association between SARS-CoV-2-specific TemRA CD4 T cells and soluble IL-10 levels was determined (Figure 5F,G).

## 4. Discussion

In this observational study, we integrate comprehensive innate and adaptive immunophenotyping, SARS-CoV-2-specific cell response, and quantitative analysis of soluble cytokines/chemokines to characterize the immune and inflammatory profile in patients admitted to the hospital because of severe COVID-19 and 6 months after recovery, and compared them with healthy controls. Our data has allowed us to identify that these patients present immune alterations after overcoming the infection, although the clinical consequences of these alterations are still unknown. Moreover, we aimed to determine whether patients presented SARS-CoV-2-specific T-cell response at the 6-month follow-up and if response prevalence and magnitude were associated with the patient’s baseline parameters.

Our study confirms previous investigations in which most patients developed marked lymphopenia [5]. Non-recovery predicts disease progression and also correlates with high levels of proinflammatory cytokines in patients with severe or critical disease. At the hospital admission time point of the present study, the lymphopenia observed was normalized over time, probably due to the administration of antivirals combined with corticosteroids during hospitalization, promoting a decrease in viral load and T-cell recovery [13,14]. In severe patients, lymphopenia is also usually accompanied by the “cytokine storm” [15]. In this sense, we observed high soluble proinflammatory IL-33 and anti-inflammatory IL-10 levels in SARS-CoV-2 patients compared with healthy donors at baseline. This situation may be due to an attempt to balance the inflammation produced during the infection’s acute phase by the immune system, combined with treatments patients received during hospitalization [16]. After SARS-CoV-2 recovery, IL-10 levels reduced, probably due to lymphopenia restoration [17]. In contrast, IL-33 levels continue to increase, in agreement with previous studies in which it seems to not respond to glucocorticoid therapy [18]. Hepatic alterations are also described during SARS-CoV-2 infection, but all liver damage-related biomarkers normalized over time in our study. Our results confirmed previous data in which hepatic injury during SARS-CoV-2 infection is likely due to systemic inflammation and less likely mediated by a cytopathic effect directed on liver cells [19,20,21].

Regarding chemokines, we observed increased TARC and MIP-1α levels in infected subjects compared to healthy donors at baseline, involved in lymphocyte and monocyte endothelial attraction and migration [22,23]. This may explain the lymphopenia we observed in SARS-CoV-2 patients at baseline, and MIP-1α continuous increment that over time may have thrombotic clinic consequences [22]. Contrary to expectations, no differences were observed at Eotaxin and MCP-1 at baseline (involved in eosinophils and monocyte attraction, respectively) [22,24], probably due to the treatment patients were receiving during the hospitalization [25]. However, Eotaxin levels increased six months later, which may have future neurological implications [26].

NK cells and monocyte subsets play a critical role in modulating the immune response at the initial viral stage. A decreased proportion of CD16^high^ and increased distribution of CD56^neg^ NK cell subsets were found at baseline in SARS-CoV-2 infected patients. These data agree with previous research in SARS-CoV-2 infection and other chronic viral diseases, in which the NK subsets alterations try to be compensated by an increased cytokines/chemokines production [27,28], as we observed in our study with the significant correlations between the inhibitory receptor NKG2A and the maturation CD57 markers’ expression and soluble cytokines/chemokines at baseline. Additionally, NK cells from the SARS-CoV-2 group also showed an upregulation of the inhibitory receptor CD158b, indicating a reduction of NK cells’ antiviral activity [29]. With respect to monocytes, their dysregulated functions are involved in acute inflammation, promoting “cytokine storm” and organs damages [30]. Our findings provide evidence to support the aforementioned data, as demonstrated by the correlations we have presented between the subset distribution and activation and endothelial adhesion markers’ expression and the levels of proinflammatory cytokines/chemokines levels, which can be considered as a clinical manifestation of that dysregulation leading to intensifying inflammation levels [31,32]. Notably, innate immune cell components were only partially restored after 6 months.

Regarding the adaptive immune response, T cells play a critical role in viral clearance. In addition to lymphopenia previously described during hospitalization, we observed high TemRA and EM subsets of CD4 and CD8 T cells in SARS-CoV-2 patients, which remained increased after six months after infection recovery. These findings suggest pronounced differentiation during virus infection and an exhausted immune response [33,34,35], similar to what occurs in other chronic viral infections [36,37]. T cells from SARS-CoV-2 patients presented a generalized overexpression of activation and exhaustion markers, indicating impaired T-cell function and promoting exacerbated activation that may also reduce host antiviral immunity, as previously reported [38]. Prolonged activation can lead to cellular exhaustion, which is characterized by high expression of inhibitory and exhaustion receptors and a different transcriptional state that is different from functional T cells [39]. Our proposal entails the identification of exhaustion hallmarks as potential biomarkers with dual utility: (1) as predictors of COVID-19 severity and immune recovery, and (2) as targets for therapeutic drug development aimed at mitigating COVID-19 symptoms, both during acute infection and post-recovery from SARS-CoV-2 [40].

We conducted an analysis of the immune memory to SARS-CoV-2 6 months after infection by examining the specific T-cell response. We observed detectable T-cell responses in all patients, across all CD4 and CD8 T-cell memory subsets, consistent with previous findings in severe patients where stronger SARS-CoV-2-specific T-cell responses may result from higher viral loads due to an inadequate early T-cell response that failed to control the virus and a dysregulated innate immunity [41]. Interestingly, we found higher levels of response in TemRA and EM CD4 and CD8 T-cells subsets, respectively, which would be in line with previous studies [41,42,43]. These results support the potential pathogenic role of CD4 T-cell responses and the protective role of CD8 T-cell responses in recovered patients [41]. We also found associations with clinical data, where patients with preserved liver profile and high anti-inflammatory cytokine levels, such as IL-10 at baseline, developed better SARS-CoV-2-specific T-cell response 6 months later [44]. These associations may be crucial in determining a strong long-term SARS-CoV-2 response and avoiding potential reinfections.

The main limitation of the study is that data on some immune markers were only available for a subset of the enrolled patients due to the multicentre nature of the study. However, despite this limitation, our finding on the longitudinal evolution of the immune response in severe SARS-CoV-2 patients who required hospitalization provides translational information for interpreting the complex innate and adaptive immune responses against this virus and for optimizing future clinical management and treatments in this life-threatening infection. Further studies are needed to determine if the persistent immune cell alterations and exhausted profile described here may lead to what is known as “long COVID syndrome” [45].

## 5. Conclusions

Our findings contribute to the immunological profiling of patients with severe SARS-CoV-2 infection, who exhibit altered biochemical parameters, increased levels of soluble cytokines and chemokines, enrichment in Effector and Terminally Differentiated T-cell memory subsets, hyperactivated and exhausted T and NK cells compared to healthy donors. In the short-term follow-up, six months after recovery, all patients showed high SARS-CoV-2-specific T-cells response, suggesting its possible association with the prior episode of severe infection, and the magnitude response was found in TemRA CD4 T-cell and EM CD8 T-cell memory subsets. However, after six months, the exhausted phenotype persists, which may pose a risk for reinfections and the development of other pathologies.

## Figures and Tables

**Table 1:** Characteristics of the SARS-CoV-2 patients.

Parameters	Values
SARS-CoV-2 subjects—n	64
Age (years)	57 (25–98)
Sex (female sex)—n (%)	23 (36.0)
Time of hospitalization (days)	7 (1–23)
Time of symptoms (days)	16 (8–38)
Pneumonia—n (%)	59 (92.2)
Deaths—n (%)	6 (9.4)
Comorbidities—n (%)	
Hypertension	15 (27.8)
Diabetes	7 (11)
Treatment during hospitalization—n (%)	
Oxygen therapy	64 (100)
Kaletra + Hydroxycloroquine + Azithromycin	29 (45.4)
Remdesivir + Dexamethasone + Heparin	33 (51.6)
Clinical data at admission	
Hemoglobin (g/dL)	13.7 (8–19)
Platelets (cells/mm^3^)	204 (104–459)
Lymphocytes (cells/mm^3^)	1114 (140–3628)
Monocytes (cells/mm^3^) ^a^	450 (120–1170)
Neutrophils (cells/mm^3^) ^a^	3236 (831–9631)
Creatinine (mg/dL) ^b^	0.8 (0.4–1.4)
D-dimer (ng/mL) ^b^	460 (140–2500)
PCR (mg/L) ^b^	37.3 (0.3–376.5)

Values are taken at baseline. Categorical variables are expressed as numbers and percentages (%). Percentages have been rounded per convention. Continuous variables are expressed as median and interquartile range (IQR). ^a,b^ Data only available from 54 and 58 patients, respectively. Abbreviations: SARS-CoV-2, severe acute respiratory syndrome coronavirus 2; PCR, C-reactive protein.

## Data Availability

The datasets presented in this study are available upon reasonable request from the corresponding author.

## Supplementary Materials

The following supporting information can be downloaded at: https://www.mdpi.com/article/10.3390/jcm12103539/s1, Figure S1: Schematic diagram of the gating strategy from a SARS-CoV-2 patient; Figure S2: Associations between soluble pro/anti-inflammatory cytokine and chemokine levels and biochemical and clinical parameters in SARS-CoV-2 patients at baseline; Figure S3: Correlations between soluble cytokine and chemokine levels and activation, maturation, inhibition, and endothelial adhesion markers in NKs and monocytes at baseline and 6 months later; Figure S4: Correlations between soluble cytokine and chemokine levels and activation and exhaustion markers in CD4 and CD8 T cells at baseline and 6 months later; Table S1: Description of T cells, NK cells, and monocytes panels analyzed by multiparametric flow cytometry; Table S2: Pro/anti-inflammatory soluble cytokine and chemokine levels in SARS-CoV-2 patients at baseline and 6 months later and healthy donors ; Table S3: Percentage of total NKs, monocytes, and T-cells subset distribution in SARS-CoV-2 patients at baseline and 6 months later and healthy donors.
