# Supplementary material for: Persistent Exhausted T-Cell Immunity after Severe COVID-19: 6-Month Evaluation in a Prospective Observational Study
Source: J Clin Med. 2023 May 18;12(10):3539. doi: 10.3390/jcm12103539 (PMC10219183; doi:10.3390/jcm12103539)
Supplement: Supplementary file 1 [file jcm-12-03539-s001.zip › jcm-2348059-supplementary.pdf]

SUPPLEMENTARY MATERIALS

Persistent Exhausted T-Cell Immunity after Severe COVID-19: 6-Month Evaluation in a Prospective Observational Study

Elena Vazquez-Alejo [1,2,†], Laura Tarancon-Diez [1,2,†], Maria de la Sierra Espinar-Buitrago [1,2], Miguel Genebat [3], Alba Calderón [3], Guillermo Pérez-Cabeza [4], Esmeralda Magro-Lopez [1,2], Manuel Leal [5,‡] and Mª Ángeles Muñoz-Fernández [1,2,*,‡]

Supplementary Table S1. Description of T cells, NK cells and monocytes panels analysed by multiparametric flow cytometry.

Panel	Antibody	Clone	Reference And Trading house	Isotype	Reference And Trading house
T cell activation	LIVE/DEAD fixable Aqua Blue Dead Cell Stain		L34966- Life Technologies		
	CD14-BV510	MOP9	563079 - BD Biosciences		
	CD19-BV510	SJ25C1	562947 - BD Biosciences		
	CD56-BV510	NCAM16.2	563041 - BD Biosciences		
	CD3-APC-Cy7	SK7	557832 - BD Biosciences		
	CD8-PB	RPA-T8	344718 - Biolegend		
	CD45RA-ECD	2H4	B49193 - Beckman Coulter		
	CD27-PerCP-Cy5.5	M-T271	560612 - BD Biosciences		
	CD38-FITC	HB7	555459 - BD Biosciences		
	CD137-PE	4B4-1	555956 - BD Biosciences	Ms IgG1,k-PE	981804 - Biolegend
	HLA-DR-APC	GRB-1	HLADRA-100T - Beckman Coulter		
	CD154-PeCy7	24-31	310832 - Biolegend	Ms IgG1,k-PE-Cy7	400126 - Biolegend
T cell exhaustion	LIVE/DEAD fixable Aqua Blue Dead Cell Stain		L34966 - Life Technologies		
	CD14-BV510	MOP9	563079 - BD Biosciences		
	CD19-BV510	SJ25C1	562947 - BD Biosciences		
	CD56-BV510	NCAM16.2	563041 - BD Biosciences		
	CD3-APC-Cy7	SK7	557832 - BD Biosciences		
	CD4-APC-R700	RPA-T4	564975 - BD Biosciences		
	CD45RA-ECD	2H4	B49193 - Beckman Coulter		
	CD27-PerCP-Cy5.5	M-T271	560612 - BD Biosciences		
	CD57-FITC	NC1	B49188 - Beckman Coulter		

	TIM-3-PE	7D3	563422 - BD Biosciences	Ms IgG1,k-PE	981804 - Biolegend
	LAG-3-Pe-Cy7	7H2C65	369310 - Biolegend	Ms IgG1,k-PE-Cy7	400126 - Biolegend
	TIGIT-AF647	A15153G	372724 - Biolegend	Ms IgG2a,k-AF647	400234 - Biolegend
	PD1-BV421	EH12.1	562516 - BD Biosciences	Ms IgG1,k-BV421	400158 - Biolegend
**T reg cells**	LIVE/DEAD fixable Aqua Blue Dead Cell Stain		L34966 - Life Technologies		
	CD3-PerCP-Cy5.5	SK7	332771 - BD Biosciences		
	CD4-APC-Cy7	OKT4	317418 - Biolegend		
	CD57-FITC	NC1	B49188 - Beckman Coulter		
	CD31-AF647	WM59	561654 - BD Biosciences	Ms IgG1,k-AF647	400130 - Biolegend
	CD127-PeCy7	R34.34	A64618 - Beckman Coulter		
	CD25-BV421	9F10	302630 - Biolegend	Ms IgG1,k-BV421	400158 - Biolegend
	FoxP3-PE	259D/C7	12-4776-42 - BD Biosciences	Ms IgG1,k-PE	981804 - Biolegend
**NK cells**	LIVE/DEAD fixable Aqua Blue Dead Cell Stain		L34966 - Life Technologies		
	CD3-BV510	SK7	564713 - BD Biosciences		
	CD14-BV510	MOP9	563079 - BD Biosciences		
	CD19-BV510	SJ25C1	562947 - BD Biosciences		
	CD56-APC-Cy7	NCAM16.2	318332 - Biolegend		
	CD16-PerCP-Cy5.5	3G8	560717 - BD Biosciences		
	CD158b-PE-Cy7	DX27	339512 - Biolegend	Ms IgG1,k-PE-Cy7	400126 - Biolegend
	HLA-DR-APC	GRB-1	HLADRA-100T - Beckman Coulter		
	CD57-FITC	NC1	B49188 - Beckman Coulter		
	TIM-3-PE	7D3	563422 - BD Biosciences	Ms IgG1,k-PE	981804 - Biolegend
	NKG2D-PECF594	1D11	562498 - BD Biosciences	Ms IgG1,k-PECF594	562292 - BD Biosciences
	NKG2A-BV421	131411	747924 - BD Biosciences	Ms IgG2a,k-BV421	400260 - Biolegend

**Monocytes**	LIVE/DEAD fixable Aqua Blue Dead Cell Stain		L34966 - Life Technologies		
	CD3-BV510	SK7	564713 - BD Biosciences		
	CD19-BV510	SJ25C1	562947 - BD Biosciences		
	CD56-BV510	NCAM16.2	563041 - BD Biosciences		
	HLA-DR-APC	GRB-1	HLADRA-100T - Beckman Coulter		
	CD16-PerCP-Cy5.5	3G8	560717 - BD Biosciences		
	CD14-APC/Cy7	MφPg	557831 - BD Biosciences		
	CD11b-FITC	ICRF44	301330 - Biolegend	Ms IgG1,k-FITC	400110 - Biolegend
	CD62L-PECF594	DREG-56	562301 - BD Biosciences	Ms IgG1,k-PECF594	562292 - BD Biosciences
	CD49d-BV421	9F10	304322 - Biolegend	Ms IgG1,k-BV421	400158 - Biolegend
	CD40-PE/Cy7	5C3	561215 - BD Biosciences	Ms IgG1,k-PE-Cy7	400126 - Biolegend
**Specific T cell response**	LIVE/DEAD fixable Aqua Blue Dead Cell Stain		L34966 - Life Technologies		
	CD14-BV510	MOP9	563079 - BD Biosciences		
	CD19-BV510	SJ25C1	562947 - BD Biosciences		
	CD56-BV510	NCAM16.2	563041 - BD Biosciences		
	CD3-PercP/Cy5.5	SK7	332771 - BD Biosciences		
	CD4-APC/Cy7	OKT4	317418 - Biolegend		
	CD45RA-ECD	2H4	B49193 - Beckman Coulter		
	CD27-PerCP-Cy5.5	M-T271	560612 - BD Biosciences		
	CD107a-FITC	LAMP-1	328606 - Biolegend		
	IL2-PE	MQ1-17H12	500307 - Biolegend		
	TNFα-APC	Mab11	502912 - Biolegend		
	IFNγ-BV421	B27	506538 - Biolegend		

Supplementary Table S2. Pro/anti-inflammatory soluble cytokine and ckemokine levels in SARS-CoV2 patients at baseline and 6 months later and healthy donors.

		HD	SCV2+		*p-value*			Detection limits (pg/mL)
			Baseline	6 months	HD vs Baseline	HD vs 6 months	Baseline vs 6 months	
**Cytokines**	**IL-1β (pg/mL)**	0.01[0-0.03]	0.004[0-0.35]	0.01[0-0.7]	**0.02**	**<0.01**	0.61	0.002-7.83
	**IFN-α2 (pg/mL)**	0.004[0.003-0.01]	0.008[0-0.03]	0.01[0-0.05]	0.17	**<0.01**	0.17	0.002-10.00
	**IFN-γ (pg/mL)**	0.03[0-0.06]	0.03[0-0.14]	0.01[0-0.13]	0.46	**0.04**	0.18	0.004-8.56
	**TNF-α (pg/mL)**	0.01[0-0.05]	0.01[0-1.12]	0.007[0-0.08]	0.18	**<0.01**	0.13	0.003-9.25
	**MCP-1 (pg/mL)**	0.3[0.1-0.7]	0.35[0-0.83]	0.47[0-2.86]	0.26	**0.03**	0.35	0.009-10.00
	**IL-6 (pg/mL)**	0.01[0-0.05]	0.02[0-1]	0.01[0-0.1]	0.58	0.16	0.06	0.007-16.60
	**IL-8 (pg/mL)**	0.03[0-0.14]	0.004[0-0.14]	0.01[0-2.73]	**<0.01**	**<0.01**	**<0.01**	0.004-6.56
	**IL-10 (pg/mL)**	0.01[0-0.6]	0.02[0-0.25]	0.01[0-0.06]	0.14	0.2	**0.01**	0.004-10.30
	**IL12-p70 (pg/mL)**	0.01[0-0.16]	0.006[0-0.05]	0.01[0-0.04]	0.18	0.06	0.35	0.002-9.30
	**IL-17A (pg/mL)**	0.001[0-0.04]	0.001[0-0.05]	0.00[0-0.008]	**0.02**	**<0.01**	**<0.01**	0.001-2.70
	**IL-18 (pg/mL)**	1.6[0.02-6.4]	2.03[0-11.2]	1.73[0-8.08]	0.51	0.75	0.9	0.01-10.35
	**IL-23 (pg/mL)**	0.04[0.01-0.09]	0.032[0-0.13]	0.03[0-0.25]	0.47	0.4	0.6	0.005-9.88
	**IL-33 (pg/mL)**	0.08[0.02-0.54]	0.08[0-0.82]	0.25[0-1.6]	0.25	**0.001**	0.1	0.01-24.60
**Chemokines**	**IL-8 (pg/mL)**	0.14[0.007-1.28]	0.17[0-0.43]	0.15[0-0.34]	0.87	0.93	0.91	0.004-2.10
	**IP-10 (pg/mL)**	0.12[0.05-0.6]	0.11[0.06-0.54]	0.14[0.06-0.85]	0.91	0.83	0.81	0.001-4.05
	**Eotaxin (pg/mL)**	0.21[0.03-0.52]	0.2[0.04-0.52]	0.3[0-1.52]	0.3	**<0.01**	**0.01**	0.01-4.25
	**TARC (pg/mL)**	0.12[0.03-0.5]	0.24[0.07-0.6]	0.14[0-0.32]	**<0.01**	0.07	**<0.01**	0.002-4.47
	**MCP-1 (pg/mL)**	0.24[0.08-0.62]	0.2[0.05-0.9]	0.14[0-0.22]	0.37	**<0.01**	**<0.01**	0.005-5.67
	**MIP-1α (pg/mL)**	0.15[0-0.53]	0.5[0-3]	0.7[0-2.6]	**<0.01**	**<0.01**	**<0.01**	0.04-27.34
	**MIG (pg/mL)**	0.35[0.05-1.05]	0.17[0.05-1.84]	0.08[0.04-0.27]	**0.03**	**<0.01**	**<0.01**	0.004-1.56
	**ENA-78 (pg/mL)**	0.01[0-0.44]	0.18[0-0.69]	0.25[0.13-1.48]	**<0.01**	**<0.01**	**<0.01**	0.001-3.25
	**MIP-3α (pg/mL)**	0.02[0-0.18]	0.04[0-0.3]	0.07[0-0.46]	**0.04**	**<0.01**	**<0.01**	0.001-1.85
	**GROα (pg/mL)**	0.01[0-0.3]	0.08[0-0.6]	0.13[0.05-0.8]	**<0.01**	**<0.01**	**<0.01**	0.004-2.72
	**ITAC (pg/mL)**	0.04[0.02-0.15]	0.07[0-0.3]	0.07[0-3.3]	**<0.01**	**<0.01**	0.83	0.001-3.21
	**MIP-1β (pg/mL)**	0.003[0-0.05]	0.01[0-0.08]	0.02[0-0.05]	**<0.01**	0.45	0.11	0.001-1.85

Values are taken at baseline and 6 months later. Continuous variables are expressed as median and interquartile ranges [IQR]. Mann-Whitney U-test was used for groups' comparisons. Wilcoxon test was conducted to compare paired events. SCV2+, SARS-CoV2 patients' group; HD, Healthy Donors' group. P-values <0.05 are highlighted

Supplementary Table S3. Percentage of total NKs, monocytes, and T-cells subset distribution in SARS-CoV2 patients at baseline and 6 months later and healthy donors.

			HD	SCV2+		*p-value*		
				Baseline	6 months	HD vs Baseline	HD vs 6 months	Baseline vs 6 months
**NK cells**	**$CD16^{high}$ NK cells**	**%$CD16^{high}$ total cells**	31[8-44]	24[3-53]	25[5-49]	0.07	0.41	0.35
		**%CD57+$CD16^{high}$ NK cells**	36[16-53]	31[7-66]	45[20-71]	0.15	**<0.01**	0.02
		**%CD158b+$CD16^{high}$ NK cells**	21[9-43]	31[6-55]	24[4-49]	0.05	0.37	0.29
		**%HLA-DR+$CD16^{high}$ NK cells**	9[3-21]	11[1-53]	7[1-53]	0.37	**0.04**	0.29
		**%NKG2A+$CD16^{high}$ NK cells**	36[13-66]	36[2-58]	3[1-32]	0.97	**<0.01**	**<0.01**
		**%NKG2D+$CD16^{high}$ NK cells**	26[17-80]	25[2-54]	5[0-21]	0.27	**<0.01**	**<0.01**
		**%TIM3+$CD16^{high}$ NK cells**	94[86-97]	87[66-98]	97[91-99]	**<0.01**	**<0.01**	**<0.01**
		**%CD57+TIM3+$CD16^{high}$ NK cells**	34[15-48]	27[6-66]	45[19-70]	0.06	**<0.01**	**0.02**
	**$CD56^{neg}$ NK cells**	**%$CD56^{neg}$ total cells**	5[1-15]	6[2-19]	8[3-14]	0.23	0.88	**0.03**
		**%CD57+$CD56^{neg}$ NK cells**	26[7-45]	23[5-67]	29[10-51]	0.88	0.27	0.49
		**%CD158b+$CD56^{neg}$ NK cells**	7[1-29]	15[1-53]	4[1-23]	**0.02**	**<0.01**	0.12
		**%HLA-DR+$CD56^{neg}$ NK cells**	47[19-68]	49[6-85]	9[2-36]	0.12	**<0.01**	**<0.01**
		**%NKG2A+$CD56^{neg}$ NK cells**	25[9-46]	29[1-57]	2[0-21]	0.17	**<0.01**	**<0.01**
		**%NKG2D+$CD56^{neg}$ NK cells**	16[6-70]	17[1-32]	4[0-19]	0.90	**<0.01**	**<0.01**
		**%TIM3+$CD56^{neg}$ NK cells**	82[71-93]	86[70-97]	83[68-89]	0.06	0.25	0.90
		**%CD57+TIM3+$CD56^{neg}$ NK cells**	22[6-42]	22[4-66]	26[9-46]	0.60	0.34	0.39
	**$CD56^{dim}$ NK cells**	**%$CD56^{dim}$ total cells**	67[47-89]	66[34-84]	74[59-88]	0.46	**0.01**	0.17
		**%CD57+$CD56^{dim}$ NK cells**	40[13-64]	31[2-61]	38[14-49]	0.18	0.27	0.42
		**%CD158b+$CD56^{dim}$ NK cells**	13[6-27]	18[6-45]	14[3-35]	0.16	0.22	0.51
		**%HLA-DR+$CD56^{dim}$ NK cells**	18[6-61]	27[8-67]	23[10-71]	**0.02**	0.60	**0.03**
		**%NKG2A+$CD56^{dim}$ NK cells**	29[7-50]	26[4-49]	7[2-31]	0.73	**<0.01**	**<0.01**
		**%NKG2D+$CD56^{dim}$ NK cells**	19[7-69]	18[2-41]	4[0-16]	0.27	**<0.01**	**<0.01**
		**%TIM3+$CD56^{dim}$ NK cells**	88[66-94]	87[78-94]	82[70-93]	0.16	0.05	**<0.01**
		**%CD57+TIM3+$CD56^{dim}$ NK cells**	37[12-61]	27[2-59]	34[12-45]	0.09	0.29	0.44
	**$CD56^{high}$ NK cells**	**%$CD56^{high}$ total cells**	5[2-17]	6[2-20]	6[2-12]	0.84	0.97	0.98
		**%CD57+$CD56^{high}$ NK cells**	6[1-27]	8[2-33]	7[2-23]	0.69	0.07	0.39
		**%CD158b+$CD56^{high}$ NK cells**	17[7-58]	37[7-86]	17[8-76]	**<0.01**	0.11	0.51
		**%HLA-DR+$CD56^{high}$ NK cells**	57[18-88]	49[11-86]	38[15-92]	0.40	0.19	0.12
		**%NKG2A+$CD56^{high}$ NK cells**	56[11-80]	49[11-91]	14[5-46]	0.49	**<0.01**	**<0.01**
		**%NKG2D+$CD56^{high}$ NK cells**	35[8-72]	32[6-72]	9[0-30]	0.18	**<0.01**	**<0.01**
		**%TIM3+$CD56^{high}$ NK cells**	92[72-96]	94[87-98]	86[72-95]	0.12	**<0.01**	**<0.01**

		**%CD57+TIM3+CD56^{high} NK cells**	6[1-26]	7[2-33]	7[2-22]	0.70	0.08	0.46
**Monocytes**	**Total monocytes**	**%Total monocytes**	41[21-69]	27[8-76]	26[16-44]	**<0.01**	**<0.01**	0.5
		**%CD11b+ total monocytes**	98[7-100]	97[2-100]	95[83-99]	0.32	0.07	0.52
		**%CD40+ total monocytes**	48[0-78]	43[0-97]	61[31-97]	0.99	0.07	**<0.01**
		**%CD49d+ total monocytes**	35[0-67]	31[0-80]	46[32-66]	0.96	**0.01**	**<0.01**
		**%CD62L+ total monocytes**	14[3-40]	13[2-85]	5[1-16]	0.80	0.24	**0.01**
	**Classical monocytes**	**%CD16^{neg}CD14^{high} monocyte cells**	75[22-90]	57[6-92]	72[40-87]	0.07	0.1	0.38
		**%CD11b+ CD16^{neg}CD14^{high}**	99[5-100]	99[2-100]	99[99-100]	0.21	0.3	0.55
		**%CD40+ CD16^{neg}CD14^{high}**	49[0-85]	61[0-99]	45[8-92]	0.32	0.53	0.52
		**%CD49d+ CD16^{neg}CD14^{high}**	46[0-73]	49[0-84]	50[29-73]	0.54	0.23	0.29
		**%CD62L+ CD16^{neg}CD14^{high}**	14[3-44]	11[1-93]	4[1-9]	0.53	**0.04**	**<0.01**
	**Interme_ diate monocytes**	**%CD16^{dim}CD14^{high} monocyte cells**	8[1-30]	14[2-38]	7[5-12]	**0.02**	0.15	**<0.01**
		**%CD11b+ CD16^{dim}CD14^{high}**	99[1-100]	99[0-100]	99[82-100]	0.5	0.82	0.65
		**%CD40+ CD16^{dim}CD14^{high}**	68[0-92]	77[0-100]	53[19-96]	0.33	0.85	0.14
		**%CD49d+ CD16^{dim}CD14^{high}**	49[0-89]	46[0-95]	66[44-92]	0.45	0.4	**<0.01**
		**%CD62L+ CD16^{dim}CD14^{high}**	15[4-57]	12[0-91]	11[2-29]	0.39	0.69	0.41
	**Patrolling monocytes**	**%CD16^{high}CD14^{dim} monocyte cells**	12[0-48]	26[0-77]	17[5-51]	**0.03**	**<0.01**	0.83
		**%CD11b+ CD16^{high}CD14^{dim}**	86[0-97]	90[0-100]	82[62-97]	0.18	0.62	0.55
		**%CD40+ CD16^{high}CD14^{dim}**	54[0-70]	49[0-91]	12[1-49]	0.89	0.18	**<0.01**
		**%CD49d+ CD16^{high}CD14^{dim}**	35[0-69]	22[0-92]	50[23-89]	0.17	0.3	**<0.01**
		**%CD62L+ CD16^{high}CD14^{dim}**	15[6-60]	13[0-36]	11[1-34]	0.22	0.32	0.23
**T-cell activation**	**Total CD4 T-cells**	**%CD4+ T-cells**	24[8-32]	21[6-38]	27[18-49]	0.76	0.58	**0.01**
		**%CD154+CD4+**	5[1-11]	6[0-21]	58[25-72]	0.59	**<0.01**	**<0.01**
		**%CD137+CD4+**	3[1-9]	6[1-26]	6[1-14]	**0.02**	**<0.01**	0.65
		**%CD38+CD4+**	41[4-61]	49[13-78]	8[2-25]	0.07	**<0.01**	**<0.01**
		**%HLA-DR+CD4+**	7[3-12]	6[1-30]	8[1-84]	0.67	0.99	0.28
		**%HLA-DR+CD38+CD4+**	1[0-2]	1[0-3]	1[0-12]	0.73	0.79	0.63
	**Central Memory CD4 T-cells**	**%CD154+ CM CD4 T-cells**	1[0-3]	1[0-7]	62[27-79]	0.97	**<0.01**	**<0.01**
		**%CD137+ CM CD4 T-cells**	1[0-7]	4[0-23]	1[0-21]	**<0.01**	0.18	**0.01**
		**%CD38+ CM CD4 T-cells**	34[3-64]	42[15-83]	10[4-29]	0.07	**<0.01**	**<0.01**
		**%HLA-DR+ CM CD4 T-cells**	4[1-7]	4[1-17]	4[1-74]	0.56	0.24	0.7
		**%HLA-DR+CD38+ CM CD4 T-cells**	2[0-5]	2[0-16]	3[0-53]	0.44	0.53	0.57
	**Effector Memory CD4 T-cells**	**%CD154+ EM CD4 T-cells**	13[2-24]	11[0-40]	41[23-66]	0.97	**<0.01**	**<0.01**
		**%CD137+ EM CD4 T-cells**	3[1-11]	9[1-24]	7[2-21]	**0.01**	**<0.01**	0.78
		**%CD38+ EM CD4 T-cells**	29[1-72]	39[8-74]	13[4-32]	**<0.01**	**<0.01**	**<0.01**
		**%HLA-DR+ EM CD4 T-cells**	11[4-14]	9[2-34]	8[2-65]	0.28	0.15	0.65
		**%HLA-DR+CD38+ EM CD4 T-cells**	4[0-17]	3[1-36]	4[1-30]	0.7	0.789	0.43
	**Naïve CD4 T-cells**	**%CD154+ Naive CD4 T-cells**	2[0-9]	2[0-16]	72[38-83]	0.41	**<0.01**	**<0.01**
		**%CD137+ Naive CD4 T-cells**	4[0-10]	5[1-46]	2[0-25]	0.05	0.62	**0.01**
		**%CD38+ Naive CD4 T-cells**	42[3-72]	49[17-89]	12[4-43]	0.11	**0.03**	**<0.01**
		**%HLA-DR+ Naive CD4 T-cells**	5[2-10]	4[1-17]	4[1-78]	0.28	0.08	0.88

**T-cell activation**		**%HLA-DR+CD38+ Naive CD4 T-cells**	4[0-10]	3[1-17]	3[1-59]	0.97	0.63	0.76
	**Terminally Differentiated CD4 T-cells**	**%CD154+ TemRA CD4 T-cells**	12[3-49]	13[1-41]	47[22-65]	0.92	**<0.01**	**<0.01**
		**%CD137+ TemRA CD4 T-cells**	11[2-33]	16[3-56]	11[3-35]	0.11	0.44	0.1
		**%CD38+ TemRA CD4 T-cells**	39[1-83]	51[8-82]	16[4-40]	**0.02**	**<0.01**	**<0.01**
		**%HLA-DR+ TemRA CD4 T-cells**	9[4-21]	8[2-61]	8[1-73]	0.78	0.37	0.71
		**%HLA-DR+CD38+ TemRA CD4 T-cells**	4[0-41]	5[1-46]	3[1-39]	0.70	0.06	0.07
	**Total CD8 T-cells**	**%CD8+ T-cells**	25[11-53]	30[7-63]	17[6-63]	0.79	0.22	0.05
		**%CD154+CD8+**	4[1-7]	7[1-21]	29[16-65]	0.02	**<0.01**	**<0.01**
		**%CD137+CD8+**	3[0-10]	7[2-44]	8[1-24]	**<0.01**	**<0.01**	0.55
		**%CD38+CD8+**	29[2-47]	47[4-76]	13[5-36]	**<0.01**	0.51	**<0.01**
		**%HLA-DR+CD8+**	25[3-54]	25[2-75]	16[3-84]	0.51	0.65	0.28
		**%HLA-DR+CD38+CD8+**	2[0-5]	2[0-11]	1[0-8]	0.42	0.97	0.35
	**Central Memory CD8 T-cells**	**%CD154+ CM CD8 T-cells**	1[0-4]	7[0-38]	23[14-53]	**<0.01**	**<0.01**	**<0.01**
		**%CD137+ CM CD8 T-cells**	2[0-7]	9[1-44]	10[0-20]	**<0.01**	**<0.01**	0.71
		**%CD38+ CM CD8 T-cells**	19[2-33]	33[3-69]	26[9-47]	**<0.01**	**<0.01**	0.39
		**%HLA-DR+ CM CD8 T-cells**	17[2-51]	20[1-51]	12[2-79]	0.6	0.49	0.26
		**%HLA-DR+CD38+ CM CD8 T-cells**	5[0-20]	7[0-37]	4[1-23]	0.3	0.80	0.31
	**Effector Memory CD8 T-cells**	**%CD154+ EM CD8 T-cells**	4[1-9]	0[1-23]	20[9-57]	**0.01**	**<0.01**	**<0.01**
		**%CD137+ EM CD8 T-cells**	1[0-4]	6[1-36]	11[3-27]	**<0.01**	**<0.01**	0.19
		**%CD38+ EM CD8 T-cells**	27[3-51]	43[2-79]	21[5-40]	**<0.01**	0.68	**<0.01**
		**%HLA-DR+ EM CD8 T-cells**	29[3-66]	30[5-82]	17[2-86]	0.67	0.37	0.14
		**%HLA-DR+CD38+ EM CD8 T-cells**	7[0-13]	10[0-68]	7[1-30]	0.12	0.35	0.28
	**Naïve CD8 T-cells**	**%CD154+ Naive CD8 T-cells**	1[0-4]	3[0-35]	51[22-68]	**0.05**	**<0.01**	**<0.01**
		**%CD137+ Naive CD8 T-cells**	4[0-16]	14[2-62]	15[0-35]	**<0.01**	**<0.01**	0.71
		**%CD38+ Naive CD8 T-cells**	28[2-72]	40[3-84]	35[13-56]	**0.03**	0.15	0.17
		**%HLA-DR+ Naive CD8 T-cells**	15[2-56]	16[1-47]	14[2-83]	0.99	0.83	0.39
		**%HLA-DR+CD38+ Naive CD8 T-cells**	8[0-23]	11[0-44]	10[1-32]	0.37	0.36	0.85
	**Terminally Differentiated CD8 T-cells**	**%CD154+ TemRA CD8 T-cells**	5[2-14]	12[1-32]	32[11-69]	**<0.01**	**<0.01**	**<0.01**
		**%CD137+ TemRA CD8 T-cells**	5[1-12]	13[3-70]	20[3-36]	**<0.01**	**<0.01**	0.39
		**%CD38+ TemRA CD8 T-cells**	35[2-65]	56[3-84]	32[10-58]	**<0.01**	0.21	**0.02**
		**%HLA-DR+ TemRA CD8 T-cells**	34[3-65]	32[2-86]	25[4-95]	0.99	0.59	0.48
		**%HLA-DR+CD38+ TemRA CD8 T-cells**	8[0-61]	8[0-72]	7[2-54]	0.62	0.98	0.91
		**Ratio CD4+/CD8+**	1[0-3]	1[0-5]	1[0-8]	0.13	0.23	**0.01**

**T-cell exhaustion**	**Total CD4 T-cells**	**%CD4+ T-cells**	58[38-76]	62[23-85]	57[26-85]	0.56	0.9	0.88
		**%CD57+ CD4 T-cells**	11[3-62]	10[1-46]	9[2-44]	0.75	0.8	0.74
		**%LAG3+ CD4 T-cells**	0.1[0-4]	0.2[0-2]	1[0-2]	0.81	0.07	**0.03**
		**%PD1+ CD4 T-cells**	7[3-21]	10[4-81]	52[37-79]	0.18	**<0.01**	**<0.01**
		**%TIGIT+ CD4 T-cells**	18[1-27]	13[3-77]	23[3-67]	0.84	0.46	0.97
		**%TIM3+ CD4 T-cells**	6[2-14]	8[1-30]	14[4-43]	0.07	**<0.01**	**0.01**
	**Central Memory CD4 T-cells**	**%CM CD4+ T-cells**	26[11-41]	23[5-48]	28[14-36]	0.24	0.54	0.29
		**%CD57+ CM CD4 T-cells**	4[2-7]	3[1-8]	2[1-15]	0.91	0.11	0.1
		**%LAG3+ CM CD4 T-cells**	0.3[0-8]	0.1[0-5]	0.1[0-10]	0.11	**0.04**	0.52
		**%PD1+ CM CD4 T-cells**	7[2-15]	7[2-90]	86[77-93]	0.23	**<0.01**	**<0.01**
		**%TIGIT+ CM CD4 T-cells**	11[5-44]	12[2-89]	17[8-93]	0.93	0.49	0.32
		**%TIM3+ CM CD4 T-cells**	1[0-5]	2[0-9]	3[1-7]	0.12	**<0.01**	0.57
	**Effector Memory CD4 T-cells**	**%EM CD4+ T-cells**	21[9-35]	21[10-46]	20[15-24]	0.7	0.72	0.94
		**%CD57+ EM CD4 T-cells**	9[2-26]	7[1-43]	8[2-47]	0.82	0.8	0.95
		**%LAG3+ EM CD4 T-cells**	0.1[0-4]	0.1[0-3]	0.3[0.1-2]	0.38	0.23	0.13
		**%PD1+ EM CD4 T-cells**	8[4-15]	10[4-76]	53[36-76]	0.07	**<0.01**	**<0.01**
		**%TIGIT+ EM CD4 T-cells**	13[1-40]	8[1-83]	16[1-68]	0.51	0.91	0.99
		**%TIM3+ EM CD4 T-cells**	4[0-6]	6[0-19]	10[3-28]	**0.03**	**<0.01**	**<0.01**
	**Naïve CD4 T-cells**	**%Naive CD4+ T-cells**	24[5-53]	24[5-48]	24[12-34]	0.76	0.81	0.25
		**%CD57+ Naive CD4 T-cells**	4[3-8]	5[1-19]	3[2-18]	0.87	0.27	0.08
		**%LAG3+ Naive CD4 T-cells**	0.1[0-12]	0.1[0-3]	0.1[0-4]	0.63	0.65	0.57
		**%PD1+ Naive CD4 T-cells**	6[2-34]	6[1-87]	76[69-88]	0.63	**<0.01**	**<0.01**
		**%TIGIT+ Naive CD4 T-cells**	8[4-21]	9[2-98]	16[5-90]	0.5	**<0.01**	0.13
		**%TIM3+ Naive CD4 T-cells**	7[2-23]	7[1-25]	10[4-21]	0.26	0.06	0.55
	**Terminally Differentiate CD4 T-cells**	**%TemRA CD4+ T-cells**	12[7-18]	20[8-45]	28[17-51]	**<0.01**	**<0.01**	**<0.01**
		**%CD57+ TemRA CD4 T-cells**	13[6-55]	15[2-76]	21[4-66]	0.40	0.08	0.19
		**%LAG3+ TemRA CD4 T-cells**	1[0-12]	1[0-9]	1[0-3]	0.65	0.7	0.5
		**%PD1+ TemRA CD4 T-cells**	10[6-34]	14[6-90]	59[33-83]	0.23	**<0.01**	**<0.01**
		**%TIGIT+ TemRA CD4 T-cells**	17[3-27]	13[2-89]	26[7-77]	0.70	**0.04**	**0.03**
		**%TIM3+ TemRA CD4 T-cells**	6[1-23]	8[1-26]	9[3-34]	0.25	0.07	0.91
	**Total CD8 T-cells**	**%CD8+ T-cells**	34[19-51]	31[14-74]	33[11-65]	0.88	0.87	0.69
		**%CD57+ CD8 T-cells**	31[8-63]	33[9-74]	33[8-79]	0.77	0.3	0.07
		**%LAG3+ CD8 T-cells**	0.1[0-4]	0.2[0-3]	1[0.3-3]	0.72	**0.02**	**<0.01**
		**%PD1+ CD8 T-cells**	11[4-19]	10[3-79]	53[44-71]	0.42	**<0.01**	**<0.01**
		**%TIGIT+ CD8 T-cells**	25[4-67]	21[4-82]	27[11-70]	0.7	0.26	**0.4**
		**%TIM3+ CD8 T-cells**	8[2-21]	12[3-31]	13[4-29]	**0.04**	**<0.01**	0.29
	**Central Memory CD8 T-cells**	**%CM CD8+ T-cells**	16[8-32]	14[4-32]	16[6-30]	0.17	0.25	0.69
		**%CD57+ CM CD8 T-cells**	10[4-22]	12[3-37]	8[4-17]	0.88	0.43	0.08
		**%LAG3+ CM CD8 T-cells**	0.1[0-0.5]	0.1[0-1]	0.1[0-1]	0.46	0.32	0.75
		**%PD1+ CM CD8 T-cells**	15[6-21]	18[3-88]	79[38-89]	**0.04**	**<0.01**	**<0.01**
		**%TIGIT+ CM CD8 T-cells**	23[4-52]	28[2-85]	33[2-78]	0.65	0.57	0.76
		**%TIM3+ CM CD8 T-cells**	3[1-13]	5[2-14]	7[3-17]	0.10	**<0.01**	**0.04**

	Effector Memory CD8 T-cells	**%EM CD8+ T-cells**	4[2-28]	5[1-28]	20[1-30]	0.75	**0.04**	**<0.01**
		**%CD57+ EM CD8 T-cells**	21[4-49]	25[6-68]	20[7-50]	0.26	0.22	0.97
		**%LAG3+ EM CD8 T-cells**	0.1[0-1]	0.02[0-1]	0.1[0-1]	0.11	0.23	0.56
		**%PD1+ EM CD8 T-cells**	6[3-18]	12[2-78]	55[42-86]	**0.01**	**<0.01**	**<0.01**
		**%TIGIT+ EM CD8 T-cells**	14[4-35]	19[4-92]	16[5-77]	0.41	0.6	0.37
		**%TIM3+ EM CD8 T-cells**	2[1-9]	4[1-11]	6[2-14]	.012	**<0.01**	**0.02**
	Naïve CD8 T-cells	**%Naive CD8+ T-cells**	27[10-53]	24[8-65]	14[4-31]	0.6	**<0.01**	**<0.01**
		**%CD57+ Naive CD8 T-cells**	10[4-28]	12[4-35]	9[4-19]	0.75	0.24	0.11
		**%LAG3+ Naive CD8 T-cells**	0.1[0-1]	0.01[0-0.3]	0[0-0.2]	0.17	0.08	0.79
		**%PD1+ Naive CD8 T-cells**	10[4-31]	12[1-89]	80[46-89]	0.74	**<0.01**	**<0.01**
		**%TIGIT+ Naive CD8 T-cells**	15[3-46]	16[3-95]	25[5-54]	0.78	0.32	0.26
		**%TIM3+ Naive CD8 T-cells**	13[4-34]	17[6-35]	21[9-39]	0.15	**<0.01**	0.37
	Terminally Differentiate CD8 T-cells	**%TemRA CD8+ T-cells**	8[4-15]	20[2-81]	49[22-83]	**<0.01**	**<0.01**	**<0.01**
		**%CD57+ TemRA CD8 T-cells**	36[13-63]	52[11-89]	59[8-93]	**0.03**	**<0.01**	0.07
		**%LAG3+ TemRA CD8 T-cells**	0.1[0-5]	0.2[0-3]	0.2[0-1]	0.23	0.28	0.67
		**%PD1+ TemRA CD8 T-cells**	10[4-32]	12[2-81]	62[39-89]	0.37	**<0.01**	**<0.01**
		**%TIGIT+ TemRA CD8 T-cells**	39[4-71]	48[11-97]	31[8-85]	0.13	0.44	0.06
		**%TIM3+ TemRA CD8 T-cells**	10[3-31]	14[3-37]	17[5-43]	0.16	**<0.01**	0.12
**T reg cells**		**%CD25+ CD4 T-cells**	9[1-19]	11[2-26]	9[2-65]	0.64	0.4	0.42
		**%CD31+ CD4 T-cells**	5[1-15]	5[1-26]	3[1-31]	0.5	0.75	0.31
		**%CD45RA+ CD4 T cells**	37[10-59]	39[16-75]	36[20-63]	0.5	0.95	**0.04**
		**%CD45RA- CD4 T cells**	61[36-89]	55[24-85]	61[35-79]	0.4	0.85	0.07
		**%Treg cells**	4[1-14]	3[1-16]	5[2-10]	0.84	0.18	**0.03**
		**%Treg total cells**	1[0.1-5]	1[0-4]	1[0-2]	0.48	0.79	0.24
		**%CD31+ Treg cells**	4[2-11]	4[1-40]	1[0-6]	0.74	**<0.01**	**0.02**
		**%CD57+ Treg cells**	2[0-6]	3[0-43]	2[0-33]	0.05	0.05	0.51
		**%CD127+ Treg cells**	41[12-80]	46[0-92]	64[30-92]	0.6	**0.04**	**0.01**
		**MFI CD127+ Treg cells**	10200[5000-15100]	12000[3600-16600]	8800[6800-11600]	0.3	0.97	0.15

Values are taken at baseline and 6 months later. Continuous variables are expressed as median and interquartile ranges [IQR]. Mann-Whitney U-test was used for groups' comparisons. Wilcoxon test was conducted to compare paired events. SCV2+, SARS-CoV2 patients' group; HD, Healthy Donors' group. P-values <0.05 are highlighted.

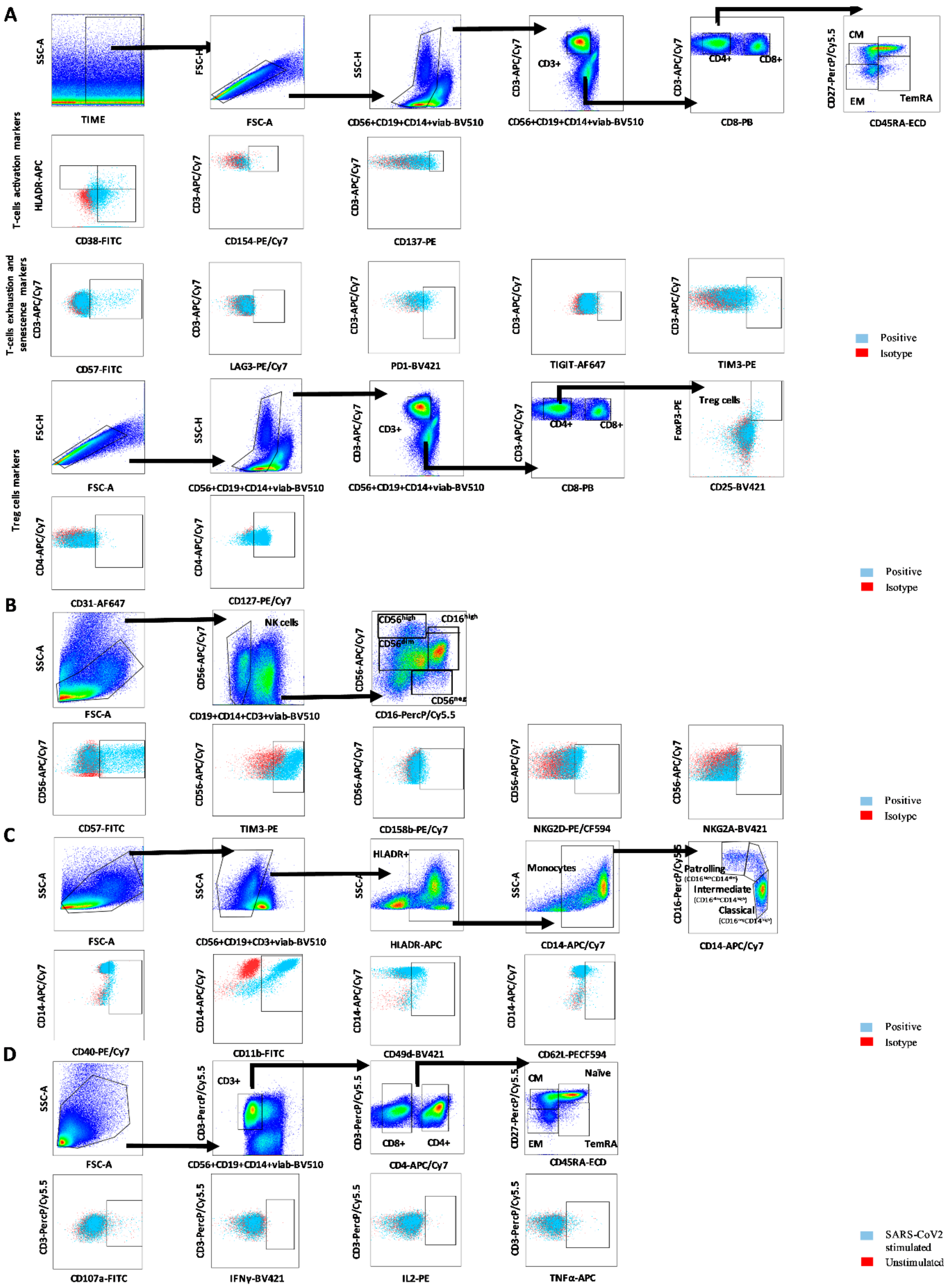

Supplementary Figure S1. Schematic diagram of the gating strategy from a SARS-CoV2 patient. T-cells and memory subsets (A), NK cells (B), monocytes (C) and SARS-CoV2 specific

T-cell and memory subsets response (D) gating strategy. Isotype controls (in red) have been used to analyze the expression of markers (in blue) for immunophenotyping. Specific SARS-CoV2 T cell response (in blue) was defined as the frequency of cells expressing intracellular cytokines and/or cytotoxicity markers after the stimulation with SARS-CoV2 peptides minus the levels of this response in the unstimulated condition (background subtraction; in red). CM: Central Memory, EM: Effector Memory, TemRA: Terminally Differentiated, Viab: Viability

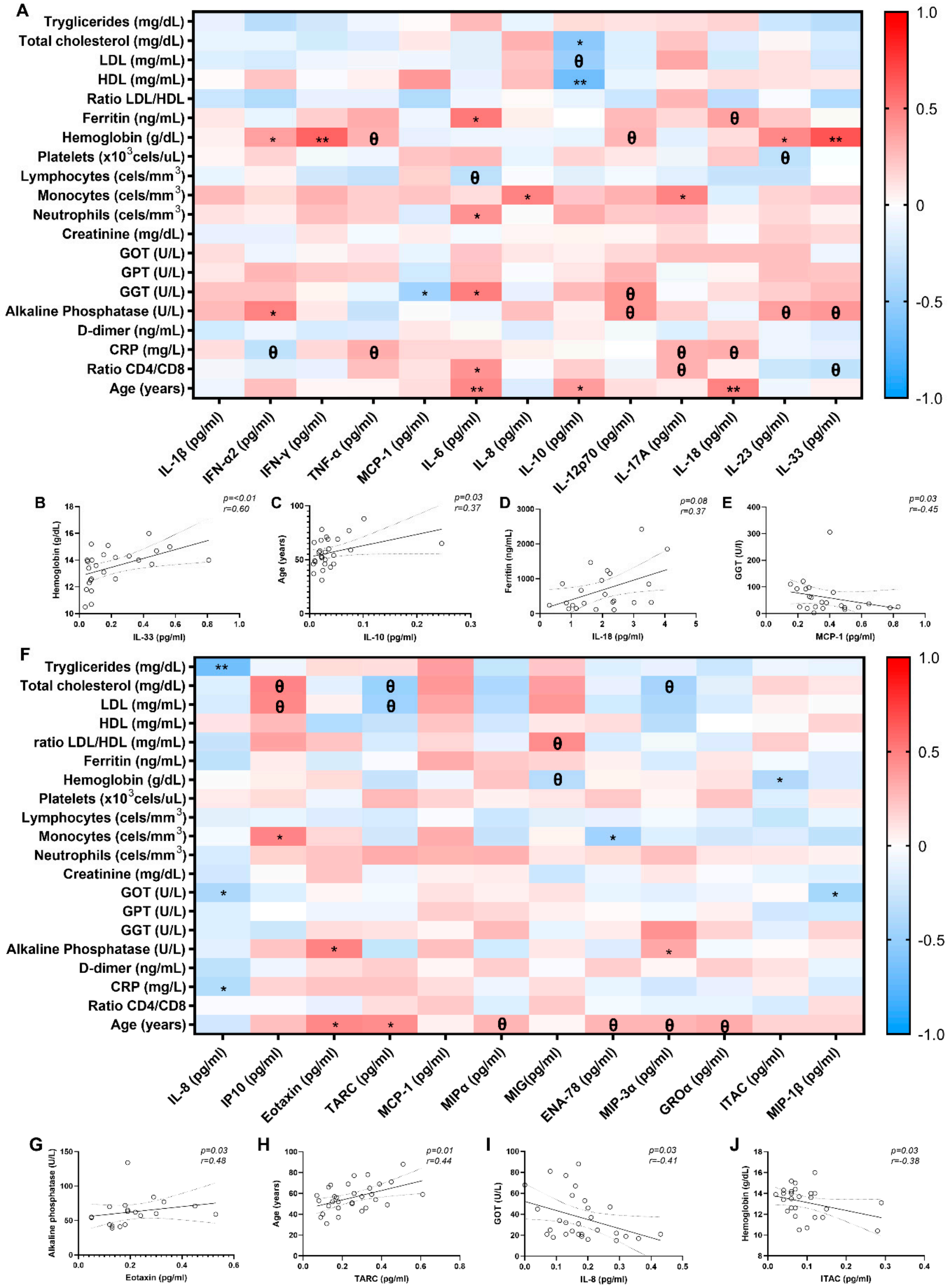

Supplementary Figure S2. Associations between soluble pro/anti-inflammatory cytokine and

chemokine levels and biochemical and clinical parameters in SARS-CoV2 patients at baseline. Correlations of cytokine levels and clinical parameters (A). Significant and close to significant associations between IL-33 and hemoglobin (B), IL-10 and age (C), IL-18 and ferritin (D), MCP-1 and hepatic enzyme Gamma Glutamyl Transferase (GGT) levels (E). Correlations of chemokine levels and clinical parameters (F). Significant associations between Eotaxin and alkaline phosphatase (data available for 20 patients) (G), TARC and age (H), IL-8 and Glutamic Oxaloacetic Transaminase (GOT) (I), ITAC and hemoglobin (J). *Data available from 32 SARS-CoV2 patients*. The Spearman ρ correlation coefficient test was used. $^{**}p \leq 0.01$, $^{*}p<0.05$, ϴ $0.05 \leq p \leq 0.1$, ns $p>0.1$.

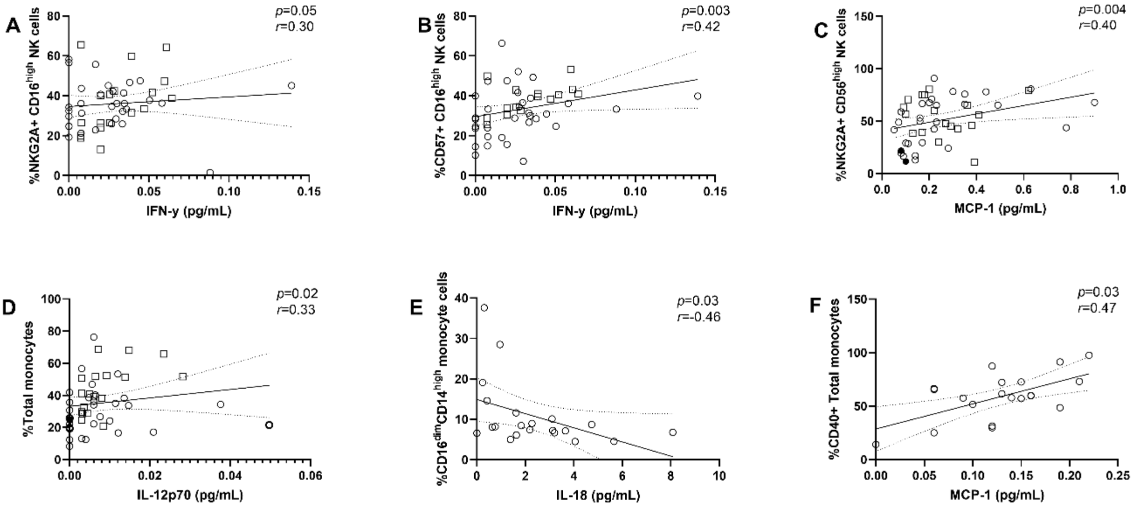

Supplementary Figure S3. Correlations between soluble cytokine and chemokine levels and activation, maturation, inhibition and endothelial adhesion markers in NKs and monocytes at baseline and 6 months later. Correlation between soluble IFN-γ and NKG2A, CD57 expression in $CD16^{high}$ NK cell subset at baseline (A, B); correlation between soluble MCP-1 and NKG2A in $CD56^{high}$ NK cell subset at baseline (C); correlation between soluble IL-12p70 and total monocytes at baseline (D); correlation between soluble IL-18 and $CD16^{dim}CD14^{high}$ monocyte subset after six months (E) and association between soluble MCP-1 and CD40 expression in total monocytes after 6 months (F). SCV2+ are highlighted with black dots and white squares represent HD. Values are taken at baseline and 6 months later. The Spearman ρ correlation coefficient test was used.

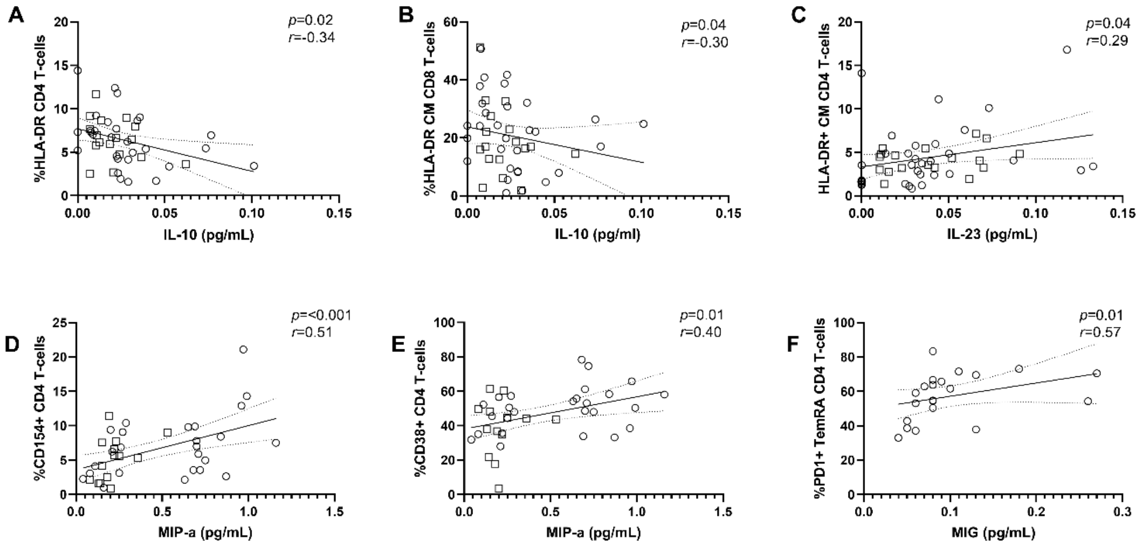

Supplementary Figure S4. Correlations between soluble cytokine and chemokine levels and activation and exhaustion markers in CD4 and CD8 T-cells at baseline and 6 months later. Association between soluble IL-10 and HLA-DR in total CD4 T-cells and Central Memory (CM) CD8 T-cells at baseline (A, B); correlation between soluble IL-23 and HLA-DR in CM CD4 T-cells at baseline (C); association between soluble MIP-α and CD154, CD38 in total CD4 T-cells at baseline (D, E); correlation between soluble MIG and PD1 in Terminally Differentiated (TemRA) CD4 T-cell memory subset 6 months later (F). SCV2+ are highlighted with black dots and white squares represent HD. Values are taken at baseline and 6 months later. The Spearman ρ correlation coefficient test was used.
